# The temporal relationship between cancer and adult onset anti-transcriptional intermediary factor 1 antibody–positive dermatomyositis

**DOI:** 10.1093/rheumatology/key357

**Published:** 2018-12-07

**Authors:** Alexander Oldroyd, Jamie C Sergeant, Paul New, Neil J McHugh, Zoe Betteridge, Janine A Lamb, William E Ollier, Robert G Cooper, Hector Chinoy, UKMyoNet, Yasmeen Ahmed, Raymond Armstrong, Robert Bernstein, Carol Black, Simon Bowman, Ian Bruce, Robin Butler, John Carty, Chandra Chattopadhyay, Easwaradhas Chelliah, Fiona Clarke, Peter Dawes, Joseph Devlin, Christopher Edwards, Paul Emery, John Fordham, Alexander Fraser, Hill Gaston, Patrick Gordon, Bridget Griffiths, Harsha Gunawardena, Frances Hall, Beverley Harrison, Elaine Hay, Lesley Horden, John Isaacs, Adrian Jones, Sanjeet Kamath, Thomas Kennedy, George Kitas, Peter Klimiuk, Sally Knights, John Lambert, Peter Lanyon, Ramasharan Laxminarayan, Bryan Lecky, Raashid Luqmani, Jeffrey Marks, Michael Martin, Dennis McGonagle, Neil McHugh, Francis McKenna, John McLaren, Michael McMahon, Euan McRorie, Peter Merry, Sarah Miles, James Miller, Anne Nicholls, Jennifer Nixon, Voon Ong, John Packham, Nicolo Pipitone, Michael Plant, Gillian Pountain, Thomas Pullar, Mark Roberts, Paul Sanders, David Scott, David Scott, Michael Shadforth, Thomas Sheeran, Arul Srinivasan, David Swinson, Lee-Suan Teh, Michael Webley, Brian Williams, Jonathan Winer

**Affiliations:** 1 Centre for Musculoskeletal Research, University of Manchester, Manchester; 2 NIHR Manchester Biomedical Research Centre, Central Manchester NHS Foundation Trust, Manchester Academic Health Science Centre, Manchester; 3 Centre for Biostatistics, University of Manchester, Manchester; 4 Arthritis Research UK Centre for Epidemiology, University of Manchester, Manchester; 5 MRC/ARUK Centre for Integrated Research into Musculoskeletal Ageing, University of Liverpool, Liverpool; 6 Department of Pharmacy and Pharmacology, University of Bath, Bath; 7 Department of Rheumatology, Royal National Hospital for Rheumatic Diseases, Royal United Hospitals Bath Foundation Trust, Bath; 8 Division of Population Health, Health Services Research and Primary Care, University of Manchester, Manchester; 9 Centre for Integrated Genomic Medical Research, University of Manchester, Manchester; 10 Department of Rheumatology, Aintree University Hospital, Liverpool, UK; 11 Department of Rheumatology, Salford Royal NHS Foundation Trust, Salford, UK

**Keywords:** dermatomyositis, myopathies, autoantibodies, epidemiology, cancer

## Abstract

**Objectives:**

To characterize the 10 year relationship between anti-transcriptional intermediary factor 1 antibody (anti-TIF1-Ab) positivity and cancer onset in a large UK-based adult DM cohort.

**Methods:**

Data from anti-TIF1-Ab-positive/-negative adults with verified diagnoses of DM from the UK Myositis Network register were analysed. Each patient was followed up until they developed cancer. Kaplan–Meier methods and Cox proportional hazard modelling were employed to estimate the cumulative cancer incidence.

**Results:**

Data from 263 DM cases were analysed, with a total of 3252 person-years and a median 11 years of follow-up; 55 (21%) DM cases were anti-TIF1-Ab positive. After 10 years of follow-up, a higher proportion of anti-TIF1-Ab-positive cases developed cancer compared with anti-TIF1-Ab-negative cases: 38% *vs* 15% [hazard ratio 3.4 (95% CI 2.2, 5.4)]. All the detected malignancy cases in the anti-TIF1-Ab-positive cohort occurred between 3 years prior to and 2.5 years after DM onset. No cancer cases were detected within the following 7.5 years in this group, whereas cancers were detected during this period in the anti-TIF1-Ab-negative cases. Ovarian cancer was more common in the anti-TIF1-Ab-positive *vs* -negative cohort: 19% *vs* 2%, respectively (*P* < 0.05). No anti-TIF1-Ab-positive case <39 years of age developed cancer, compared with 21 (53%) of those ≥39 years of age.

**Conclusion:**

Anti-TIF1-Ab-positive-associated malignancy occurs exclusively within the 3 year period on either side of DM onset, the risk being highest in those ≥39 years of age. Cancer types differ according to anti-TIF1-Ab status, and this may warrant specific cancer screening approaches.

**Rheumatology key messages**

- Anti-TIF1-Ab-positive dermatomyositis is associated with an increased risk of malignancy, particularly ovarian cancer.
- This increased risk is only present in the 3 years on either side of dermatomyositis onset.
- This significant association only exists for those ≥39 years of age.

## Introduction

The idiopathic inflammatory myopathies (IIMs) are a group of diseases characterized by skeletal muscle inflammation, usually symmetrical proximal muscle weakness, elevated skeletal muscle enzymes and the presence of circulating myositis-specific/associated autoantibodies (MSA/MAAs) [1, 2]. A strong association with cancer development has been noted in the IIMs, especially in DM [3, 4]. A meta-analysis reported that the risk of cancer development in DM is around five times higher than that in the general population. The DM cancer risk is twice that compared with the cancer risk in polymyositis, and it is well recognized that cancer risk is highest in the years prior to and following IIM onset [3].

The presence of anti-transcriptional intermediary factor 1 antibody (anti-TIF1-Ab) is DM specific and confers an even greater risk of cancer development, with the risk of cancer-associated myositis (CAM; cancer occurring within 3 years prior to or following an IIM onset) ranging from 38 to 71% [5–9]. It remains unclear whether the cancer site distribution differs according to anti-TIF1-Ab status.

Anti-TIF1-Ab is one of the most common antibodies to be detected in juvenile DM, yet here it is not associated with malignancy [10]. Fujimoto *et al. *[10] indicated a potential age cut-off for cancer development in adults with DM, whereby anti-TIF1-Ab positivity appears to confer no cancer risk for those DM patients <40 years of age. Further confirmation of this observation would help to inform clinical cancer screening. Prior studies have only followed study participants up to a maximum of 3 years following IIM onset, due in part to the arbitrary definition of CAM. Therefore the long-term risk of cancer associated with anti-TIF1-Ab positivity remains unclear.

In 2007, using data from the UK Myositis Network (UKMyoNet), Chinoy *et al. *[6] investigated the prevalence of anti-TIF1-Ab in IIM cases with and without cancer and reported that 50% of anti-TIF1-Ab-positive cases suffered from CAM. This same cohort has now been followed up for a further 10 years and additional anti-TIF1-Ab-positive/-negative participants have been recruited. Further knowledge of the longer-term risk of cancer, common sites of malignancy and age association would usefully inform cancer screening strategies and optimize patient management. The current study aimed to compare the 10 year cancer risk of anti-TIF1-Ab-positive *vs* -negative DM cases using UKMyoNet data.

## Methods

As anti-TIF1-Ab is DM specific, only DM cases were analysed. Adult DM participants (≥18 years of age at DM diagnosis) were recruited from 74 participating centres across the UK (listed in the Acknowledgements). All had confirmed probable or definite DM according to the Bohan and Peter criteria [11, 12]. The International Myositis Classification Criteria [13] score was also calculated for each case and only those with a score of at least 55% (‘probable IIM’) were included. A standardized, single-page clinical pro forma was used to collect baseline data regarding demographics and clinical features. Date of DM onset was defined as the time of symptom onset, according to the recruiting consultant.

All patients were tested for the presence of MSA/MAAs. Two 10 ml whole blood samples were taken into EDTA to enable separation of plasma by centrifugation, permitting autoantibody testing for determination of the presence of MSA anti-synthetases (anti-Jo-1, -PL7, -PL12, -EJ, -OJ, -KS, -Zo), other MSAs (anti-TIF1, -Mi-2, -SAE, -SRP, -MDA5, -NXP2, -HMGCR) and MAA (anti-PM-Scl, -Ku, -U1-RNP, -U3-RNP). All autoantibodies were detected using radio-immunoprecipitation, except anti-HMGCR, which was detected by ELISA.

Patients’ written consent to participate was obtained according to the Declaration of Helsinki, ethical approval having been gained locally at each participating centre (North West Research Multicentre Research Ethics Committee 98/8/86). Consent included permission to allow individual patient details to be registered with the UK Health and Social Care Information Centre (HSCIC) and from which future cancer diagnosis information could be obtained. UKMyoNet recruitment began in 1999 and cancer occurrence linkage was carried out until December 2017.

## Analysis

Baseline clinical variables of the anti-TIF1-Ab-positive and -negative cohorts were compared. Categorical variables were compared through chi-squared testing and non-normally distributed continuous variables through Mann–Whitney U testing.

Where a cancer diagnosis was made, the time between DM onset and cancer diagnosis was calculated and employed as the follow-up time. Where no cancer diagnosis was made, the time between DM onset and the follow-up cut-off date (December 2017) was calculated. Cases were censored at the end of their follow-up period if no cancer diagnosis was made. The proportion of each cohort that had developed a cancer within the 3 years preceding DM onset was calculated. Subsequently, using this proportion as a baseline, the cumulative incidence of cancer occurrence after DM onset was estimated according to Kaplan–Meier methods. Hazard ratios (HRs) for the time to cancer diagnosis were calculated using a Cox proportional hazard model adjusted for age, gender and smoking status and then according to anti-TIF1-Ab status.

## Results

### Cohort characteristics

Data from 263 (69% female) verified DM cases with a total of 3252 person-years follow-up were analysed (Table 1). The median follow-up time was 10 years [interquartile range (IQR) 6–15] for anti-TIF1-Ab-positive cases and 12 years (IQR 8–18) for anti-TIF1-Ab-negative cases. Fifty-five (21%) of the studied cases were anti-TIF1-Ab-positive. Of the 208 anti-TIF1-Ab-negative cases, 40 (19%) were Jo-1 positive, 15 (7%) were positive for Mi2 and 8 (4%) were positive for NXP2. A higher proportion of the anti-TIF1-Ab-positive cases were female compared with anti-TIF1-Ab-negative cases. Equal proportions of each cohort smoked. The age of DM onset was significantly older for those that subsequently developed cancer compared with those that did not (*P* < 0.05). The median age of DM onset and cancer onset were similar in the anti-TIF1-Ab-positive and -negative cohorts.

**Table 1:** Baseline demographics and time to cancer occurrence for the anti-TIF1-Ab-positive and negative cohorts

		Total cohort (*n* = 263)	Anti-TIF1-Ab positive (*n* = 55)	Anti-TIF1-Ab negative (*n* = 208)
Female, *n* (%)		181 (69)	44 (80)	137 (66)*
Smokers, *n* (%)		82 (31)	17 (31)	65 (31)
Follow-up, years, median (IQR)		10 (7–17)	10 (6–15)	12 (8–18)
				
Age of DM onset, years, median (IQR)	No subsequent cancer diagnosis	47 (35–60)	45 (34–58)	47 (37–59)
	Subsequent cancer diagnosis	59 (48–66)	59 (50–65)	58 (49–69)
Age at cancer diagnosis, years, median (IQR)		61 (52–68)	60 (52–67)	62 (52–70)
Time from DM to cancer onset, years, median (IQR)		4.3 (1.5–9.4)	1.4 (0.7–2.5)	5.0 (2.5–10.4)*
Cancer cases during total follow-up period, *n* (%)		53 (20)	21 (38)	32 (15)*
Cases classified as CAM^a^, *n* (%)		42 (16)	21 (38)	21 (10)*
Patients with cancer within 3 years preceding DM onset, *n* (%)		16 (6)	7 (13)	9 (4)*
Patients with cancer within 3 years following DM onset, *n* (%)		26 (10)	14 (26)	12 (6)*

^a^CAM: cancer occurring within 3 years either side of DM onset.

Variables of the anti-TIF1-Ab-positive cohort were compared against those of the anti-TIF1-Ab-negative cohort. *P < 0.01 (categorical variables were compared with the chi-squared test, continuous variables were compared using the Mann–Whitney U test). Anti-TIF1-Ab: anti-transcriptional intermediary factor 1 antibody.

### Cancer diagnoses during follow-up

**Fig. 1:**
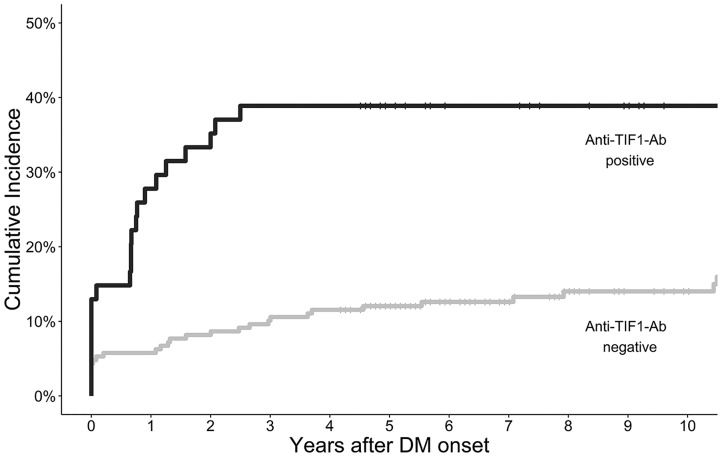
Kaplan-Meier plot of cumulative incidence of cancer onset following dermatomyositis diagnosis stratified according to anti-TIF1-Ab status Anti-TIF1-Ab: anti-transcriptional intermediary factor 1 antibody.

Fifty-three (20%) of the overall cohort were diagnosed with cancer during the entire follow-up period. An increased proportion of anti-TIF1-Ab-positive cases developed cancer throughout the study period compared with anti-TIF1-Ab-negative cases [21 (38%) *vs* 32 (15%)] (Fig. 1). The median time from DM onset to subsequent cancer diagnosis throughout the entire follow-up period was shorter for anti-TIF1-Ab-positive compared with anti-TIF1-Ab-negative cases [1.4 years (IQR 0.7–2.5) *vs* 5.0 (2.5–10.4)]. All detected cancers in the anti-TIF1-Ab-positive cases occurred within 2.5 years following DM onset; no further cases of cancer were detected within the remaining follow-up period. In contrast, new cases of cancer were detected in anti-TIF1-Ab-negative cases throughout the 3–10 year period following DM onset, with 9.8% (95% CI 7.5, 12.0) at 5 years, 11.7% (95% CI 9.2, 14.2) at 7.5 years and 14.1% (95% CI 11.2, 16.8) at 10 years.

Cox proportional hazard modelling, adjusted for age, gender and smoking status, revealed that anti-TIF1-Ab positivity was significantly associated with a shorter time between DM and cancer onset (during the entire follow-up period) [HR 3.2 (95% CI 1.8, 5.5)].

Malignancy was more frequent in older compared with younger anti-TIF1-Ab-positive cases. Fifteen anti-TIF1-Ab-positive cases were <39 years of age at DM onset and none developed cancer during the follow-up period, whereas 21 (53%) of the 40 anti-TIF1-Ab-positive cases ≥39 years of age at DM onset developed cancer during the follow-up period. Older age was significantly associated with cancer onset [HR 1.04 (95% CI 1.02, 1.07)]; the significant association between anti-TIF1-Ab positivity and early cancer onset only existed for those ≥39 years of age [HR 4.1 (95% CI 2.5, 6.7) *vs* HR 0.8 (95% CI 0.1, 6.5) for patients ≥39 and <39 years of age, respectively].

### Site of cancer

Within the anti-TIF1-Ab-positive cohort, breast cancer was the most common malignancy (33%), followed by ovarian cancer (19%) and lymphoma (14%). Breast cancer (25%), lymphoma (13%) and bowel cancer (13%) were the three most common cancers in the anti-TIF1-Ab-negative cohort. Ovarian cancer constituted a significantly higher proportion of cancers in the anti-TIF1-Ab-positive *vs* -negative cases: 19% *vs* 2%, respectively (chi-squared *P*-values <0.05). Five of the 53 (9%) cancers in the whole cohort were ovarian and 4 (80%) of these occurred in anti-TIF1-Ab-positive cases. Four of the 16 (25%) documented cancers in the anti-TIF1-Ab-positive female cohort were ovarian, compared with 1 of 26 cancers (4%) in the female anti-TIF1-Ab-negative cohort.

## Discussion

This is the first study to investigate the temporal relationship between anti-TIF1-Ab positivity and cancer onset in a large DM cohort over a 10 year follow-up period after DM onset. Anti-TIF1-Ab-positive-associated malignancy occurred exclusively within 3 years either side of the DM onset, and no incident cancer cases were detected in this group within the subsequent 7.5 years. This finding is not likely to be due to a disparity in follow-up time between anti-TIF1-Ab-positive and -negative cases, as the median follow-up times were similar for both groups: 10 years and 12 years, respectively. Further, this finding is unlikely to be due to differences in cancer detection methods, as both cohorts’ cancer diagnoses were identified through HSCIC data, ensuring capture of all incident cancers during the follow-up period. Although a number of studies have now confirmed the strong association between anti-TIF1-Ab positivity and increased malignancy risk within 3 years of IIM onset [5–8], no previous study has investigated the longer-term risks.

We have also shown that the distribution of cancer sites appears to vary according to anti-TIF1-Ab status, with female anti-TIF1-Ab-positive patients at increased risk of ovarian cancer [14, 15]. A number of studies have highlighted the increased risk of ovarian cancer associated with DM, and the current study has confirmed this finding over a 10 year longitudinal follow-up. However, this is the first large study to identify that ovarian cancer is overrepresented in anti-TIF1-Ab-positive individuals, suggesting that the true association between DM and ovarian cancer may be through possession of anti-TIF1-Abs. Clinicians should therefore apply increased vigilance for potential ovarian pathology in anti-TIF1-Ab-positive cases.

This study has identified that the significant association between anti-TIF1-Ab positivity and early cancer diagnosis exists only for those ≥39 years of age. This finding is consistent with the finding by Fujimoto *et al.* [10], who found that anti-TIF1-Ab-positive adults <40 years of age did not suffer from co-existing malignancy, whereas those >40 years of age had a high risk of cancer (75%). Our findings add strength to the hypothesis that there exists a subset of young adult anti-TIF1-Ab-positive cases who do not have a discernible increased risk of cancer, similar to that observed in TIF1-Ab-positive juvenile DM [16]. Given that older age of IIM onset is associated with CAM regardless of autoantibody positivity [15], and anti-TIF1-Ab positivity confers an increased risk, detailed screening for cancer in anti-TIF1-Ab-positive patients ≥39 years of age is advocated.

The strengths of this study include the large anti-TIF1-Ab-positive and -negative cohorts and the long-term detection of cancers through the HSCIC. Survival bias is a limitation, as patients would have to have survived sufficiently long to be recruited into UKMyoNet. Potential inaccuracy of identification of DM onset may have led to under- or overestimation of the cumulative incidence of cancer; however, this is unlikely to have impacted the substantial difference in cancer incidence between the anti-TIF1-Ab-positive and -negative cohorts. Further, the lack of data on titres of anti-TIF1-Ab and lack of serial samples have limited the ability to investigate the utility of repeat sampling. Finally, variation in sampling times throughout each patient’s disease stage and treatment course could potentially have impacted upon positivity for anti-TIF1-Ab.

A distinct approach to cancer screening according to anti-TIF1-Ab status can be advocated. It may be that a focus on screening for cancer within the first 3 years after DM onset and particularly screening for ovarian cancer in anti-TIF1-Ab-positive female patients may be required. Our findings also strengthen the hypothesis that inflammatory myopathies represent a paraneoplastic reaction initiated by attempted immune-mediated clearance of a cancer [17].

### Conclusions

This study has characterized the long-term relationship between anti-TIF1-Ab status and cancer onset. Testing for anti-TIF1-Ab in newly diagnosed DM cases can help guide cancer screening strategy. The findings that earlier cancer onset is associated with anti-TIF1-Ab positivity, particularly in those ≥39 years of age, and that more cancers are ovarian are of important clinical significance.
